# Serospatial epidemiology of zoonotic *Coxiella burnetii* in a cross section of cattle and small ruminants in northern Nigeria

**DOI:** 10.1371/journal.pone.0240249

**Published:** 2020-10-19

**Authors:** Nusirat Elelu, Adefolake Ayinke Bankole, Ramat Jummai Musa, Ismail Ayoade Odetokun, Musa Rabiu, Khalid Talha Biobaku, Abdulfatai Aremu, Akeem Olayiwola Ahmed, Mohammed Ibraheem Ghali, Mashood Abiola Raji, Ndudim Isaac Ogo, Sally Jane Cutler, Gabriel Adetunji Taiwo Ogundipe

**Affiliations:** 1 Faculty of Veterinary Medicine, University of Ilorin, Ilorin, Nigeria; 2 School of Health, Sport and Bioscience, University of East London, London, United Kingdom; 3 National Veterinary Research Institute, Vom, Nigeria; 4 Faculty of Veterinary Medicine, University of Ibadan, Ibadan, Nigeria; University of Zambia, ZAMBIA

## Abstract

The persistent and highly transmissible *Coxiella burnetii* is a neglected infection that negatively affects reproductive parameters of livestock. It is also of zoonotic importance and has been reported to cause devastating human infections globally. Domestic ruminants represent the most frequent source of human infection. Data from Nigeria are very few and outdated. There is a significant gap in up-to-date information on the exposure, spatial distribution and risk factors of infection of this important disease. The exposure to *C*. *burnetii* was determined using sensitive serological assays in cattle and small ruminants. A total of 538 animals made up of 268 cattle and 270 small ruminants were sampled from three northern Nigerian states. The proportion of cattle sampled that were seropositive from the study locations were: Kwara 14/90 (15.6%; 95% CI: 8.8–24.7); Plateau 10/106 (9.43%; 95% CI: 4.6–16.7) and Borno 4/72 (5.56%; 95% CI: 1.5–13.6) states. Lower seroprevalence was recorded among the small ruminants sampled, with positives recorded from sheep and goat sampled from only Kwara state 6/184 (3.3%; 95% CI: 1.2–7.0); while none of the small ruminants sampled from Plateau were seropositive. The results of the bivariate analysis showed that none of the tested independent variables (village, age group, sex, breed of cattle, presence of ticks, reproductive status, and management system) were statistically significant factors associated with seropositivity of cattle for antibodies to *C*. *burnetii*. Stakeholders involved in animal husbandry should be duly educated on proper disposal of birth products as well as bodily fluids in order to reduce environmental contamination, persistence and human infection.

## Introduction

*Coxiella burnetii*, is an important zoonotic pathogen affecting both animals and humans. It is a Gram-negative obligate intracellular bacterium with a worldwide distribution except in New Zealand [1, 2]. Due to the public health significance of *C*. *burnetii*, it has the double-sided risk of affecting the reproductive performance of livestock as well as the potential to infect individuals that come in contact with infected animals. The primary mode of infection is reported to be through inhalation of aerosolized bacteria shed into the environment along with birth products, urine, faeces and other fluids of infected animals where it persists due to pseudo-sporulation [3–5]. *C*. *burnetii* is a multi-host infectious agent that affects a variety of wild and domestic animals, leading to significant economic loss due to reproductive disorders such as infertility, stillbirth, abortions and weak offspring [6]. Cattle, sheep, and goats are the most common reservoir of human infection [5]. A high seroprevalence of up to 59.8% has been reported in dairy cattle and 43.2% of milk samples from Nigeria using a capillary agglutination test [7]. A more recent study reported 14.5% animal-level prevalence and a herd-level prevalence of 57.1% [8]. Studies utilizing polymerase chain reaction (PCR), to determine pathogen burden in fed ticks from Nigerian cattle showed a prevalence of 14% and 25% of *C*. *burnetii* in ticks collected from Southwestern and Northcentral regions of Nigeria, respectively [9, 10].

Human Q fever cases caused by *C*. *burnetii* infection are often related to exposure to infected animals or animal products. This further indicates that Q fever may be an important health problem in Nigeria despite very scanty evidence. Q fever has led to major epidemics in Europe involving thousands of human cases [11]. In some African counties such as Tanzania, Tunisia and Burkina Faso, *Coxiella* infection was reported to account for up to 9% of humans hospitalized for febrile illnesses [12]. A previous study carried out in Nigeria reported that 44% of blood donors had anti-*C*. *burnetii* antibodies [13]. Another recent study reported 12% seroprevalence in Northeast Nigeria [14]. The attendant effect on overall livelihood from direct economic losses and loss of man-hours due to clinical human infection is enormous. This could further impoverish the rural poor and also affect the supply of much-needed meat protein in sub-Saharan Africa.

Several studies on *C*. *burnetii* in both human and animals has been carried out in Africa [15, 16]; and as reviewed by Vanderburg et al. [12]. Presently, only a few studies exist in some Nigerian states on the current burden of *C*. *burnetii* in animals and humans, with the majority carried out in the 70s and 80s [7, 17, 18]. Despite the fact that Nigeria is the fourth largest producer of cattle in Africa and that coxiellosis is one of the diseases that negatively affects reproductive parameters leading to loss of replacement stocks, there is a significant gap in up-to-date information on the burden, spatial distribution and risk factors of infection of this important abortifacient disease. Evidence-based knowledge of the burden of *C*. *burnetii* is important in formulating control, especially because of the zoonotic risk of the disease. Moreover, knowing the disease burden in animals would indicate the potential risk to the human population especially in high-risk groups such as cattle herders. Serology is a valuable screening approach to determine exposure to infectious diseases. The Enzyme-Linked Immunosorbent Assay (ELISA) serological technique has shown usefulness in measuring disease burden in veterinary medicine and has been used successfully in several studies involving *C*. *burnetii* [19–21]. This study therefore seeks to assess the level of exposure to *C*. *burnetii* in livestock using the sensitive serological ELISA technique in northern Nigeria.

## Materials and methods

### Study sites

Ruminant husbandry system in Nigeria is considerably similar to what is obtainable in the tropics where cattle and small ruminants are generally reared under the extensive pastoral husbandry system with little supplementary feeding. The animals are herded in hundreds and are moved from the arid/semi-arid northern Nigeria to the sub-humid and lush humid southern parts of the country. The semi-intensive husbandry system is the second most common especially among small ruminant keepers whereby the animals are sheltered indoors at night for security purpose and released for grazing during the day. A few ranches exist in the country that practice the intensive system of management. These are usually government owned and rarely by high net worth individuals.

**Fig 1:**
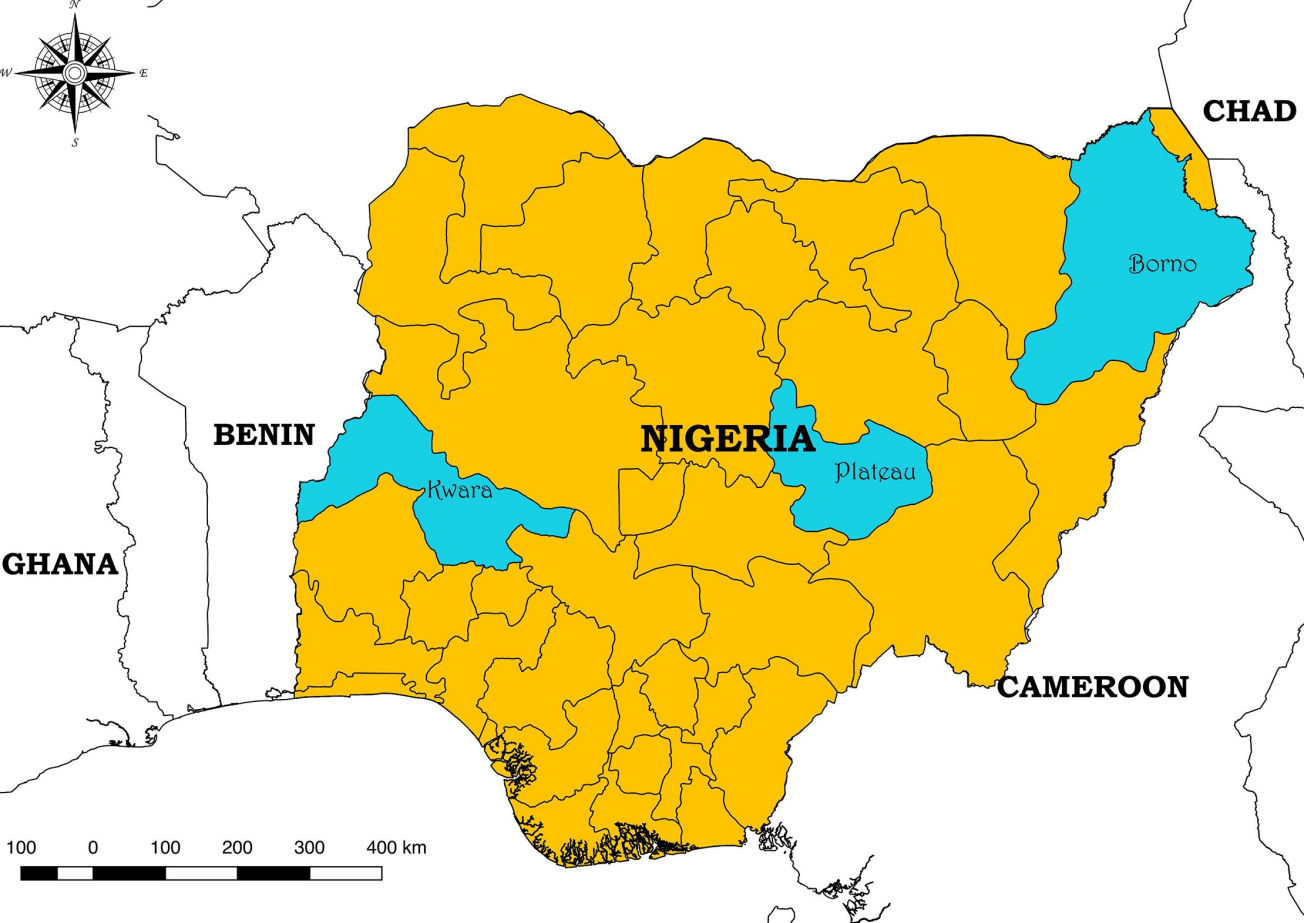
Map of Nigeria (within West Africa), showing the three northern states (Kwara, Plateau and Borno) where cattle and small ruminants were sampled.

The study was carried out in three northern Nigerian states: Kwara, Plateau and Borno (Fig 1). Kwara State comprised of rainforest in the south and wooded savannah in the larger part of the state [22]. Plateau is the highland area of central Nigeria is comprised of northern Guinea savanna vegetation, now mainly open grassland and rocky hills [23]. Borno State is located in northeastern Nigeria and contains semi-arid savannah with flooded areas toward Lake Chad [24].

### Study design and sample size estimation

A cross-sectional study design was employed to sample cattle between March to June 2019 from three states in northern Nigeria (Kwara, Plateau and Borno). Convenient sampling was used to choose nine and twelve villages in Kwara and Plateau states, while only one village was safe to be sampled in Borno State due to the security situation.

In order to estimate the seroprevalence of *C*. *burnetii*, where an expected herd prevalence (P_exp_) was 14.5% obtained from a previous study on *C*. *burnetii* carried out in Nigerian cattle [8], with a desired absolute precision (d) of 5% at a 95% confidence level. The total number of animals to be sampled was calculated from the formula: n = 1.96^2^ P_exp_ (1 –P_exp_)/d^2^ [25]. Therefore, a minimum of 190 animal each were to be sampled for cattle and small ruminants from across the three states. The cattle sampled were from nomadic pastoralists. Additional cattle samples were collected from the abattoirs in Kwara State. All the small ruminants in this study were sampled from abattoir in Kwara and Plateau States.

### Sampling collection and processing

#### Cattle

Blood samples were collected from each animal by jugular venipuncture into 10 ml vacutainer tubes. Samples were labelled and put into a cool box with ice while in the field. The age, sex, management system and the reproductive status of each animal were recorded. Animals were aged in years based on visual assessment and information obtained from livestock herders. We also determined the presence of ticks on each animal sampled by examining the head, neck, ventrum, dorsum, perineum/prepuce, scrotum/udder, tail and legs.

#### Small ruminants

Small ruminants were sampled from those slaughtered in abattoirs in both Kwara and Plateau States. However, no small ruminant samples were collected from Borno State due to insecurity of its environs. Blood samples were randomly collected from slaughtered small ruminants into sterile universal bottles after their jugular veins were severed. Animals were then examined to determine their age, sex, species and presence of ticks. All the samples were then promptly transported via cold chain to the laboratory.

### Blood processing

At the laboratory, the blood samples were centrifuged at 2500 revolutions per minute (rpm) for ten minutes for serum separation. The clear supernatants formed were then transferred into labelled plain bottles and stored at -20˚C until used for further analysis.

### Serological assay

We used ELISA to screen all collected samples for immunoglobulin G (IgG) antibodies to C. burnetii using an IDEXX Q-Fever (Coxiella burnetii) Antibody Test Kit (Idexx Laboratories, Westbrook, ME, USA) following manufacturer’s instructions. The plates were read at a wavelength of 450 nm in a microplate reader (BioTek 800TS, USA). The results were expressed as percent positivity (PP) as follows:

$$\%positivity=\frac{Sample\;OD_{450}-Negative\;Control\;OD_{450}}{Positive\;Control\;OD_{450}-Negative\;Control\;OD_{450}\;X\;100\%}$$

OD–Optical density.

Animals were classified as negative if the percentage positivity was <30%, suspect if between 30 and 40% and positive if ≥ 40%. Suspect sera were retested, and if unresolved, they were included as negatives in the data analysis.

### Mapping

Each village sampled was georeferenced using a handheld GPS device (Garmin eTrex). The locations were mapped using QGIS version version 2.18. For mapping purposes, a proportional circle map was generated to show percentage *Coxiella* antibody seropositivity by sampled location.

### Data analysis

Each animal was assigned a unique identifier based on the herd identifier and the order of sampling. Data were entered into Microsoft Excel for clean-up and SPSS version 26 (SPSS for Mac, Version 26, SPSS Inc., Chicago, IL, USA) was utilized for statistical analysis. The prevalence at animal-level was separately calculated in cattle and small ruminants. Descriptive statistics was carried out for seroprevalence result and individual animal data. To estimate the confidence intervals for seroprevalence, the Clopper-Pearson exact interval method [26] was used. The association between potential risk factors (independent variables: village, age group, sex, breed of cattle, presence of ticks, reproductive status, and management system) and the individual animal serological status were assessed using the bivariate analysis (Chi-squared test or Fishers’ exact test for cells with values less than five). Serological status for *C*. *burnetii* was considered as a binary outcome, either positive or negative. Associations were not assessed for Plateau and Borno states due to incomplete data. The level of significance was set at P < 0.05.

### Ethical approval

Ethical approval for the study was granted by the University of Ilorin ethical review committee (Approval number: UERC/ASN/2018/1387) and the Kwara State Ministry of Agriculture. We also obtained voluntary informed consent from livestock herders and abattoir workers.

## Results

During the period of study, nine and five cattle keeping villages from three local government areas (LGA) each were studied from Kwara and Plateau States, respectively. An additional 20 cattle were sampled from the abattoir in Kwara State. The security situation of Borno State restricted the number of cattle sampled, thus only one village from one LGA was safe to sample. A total of 538 animals made up of 268 cattle and 270 small ruminants were sampled from three northern Nigerian states: Kwara, Plateau and Borno. A breakdown of the animals sampled is presented in Table 1.

**Table 1:** The distribution of cattle and small ruminants sampled from three northern Nigerian states.

State	LGA/Location	Number of cattle sampled	Number of small ruminants sampled
Kwara			
	Asa	21	
	Ilorin East	8	
	Ilorin South	41	
	Abattoir	20	
	Sub-total	90	184
Plateau			
	Barkin Ladi	41	
	Bokkos	36	
	Jos North	29	
	Sub-total	106	86
Borno			
	Jere	72	
	Sub-total	72	0
**Total**		**268**	**270**

### Spatial distribution

**Fig 2:**
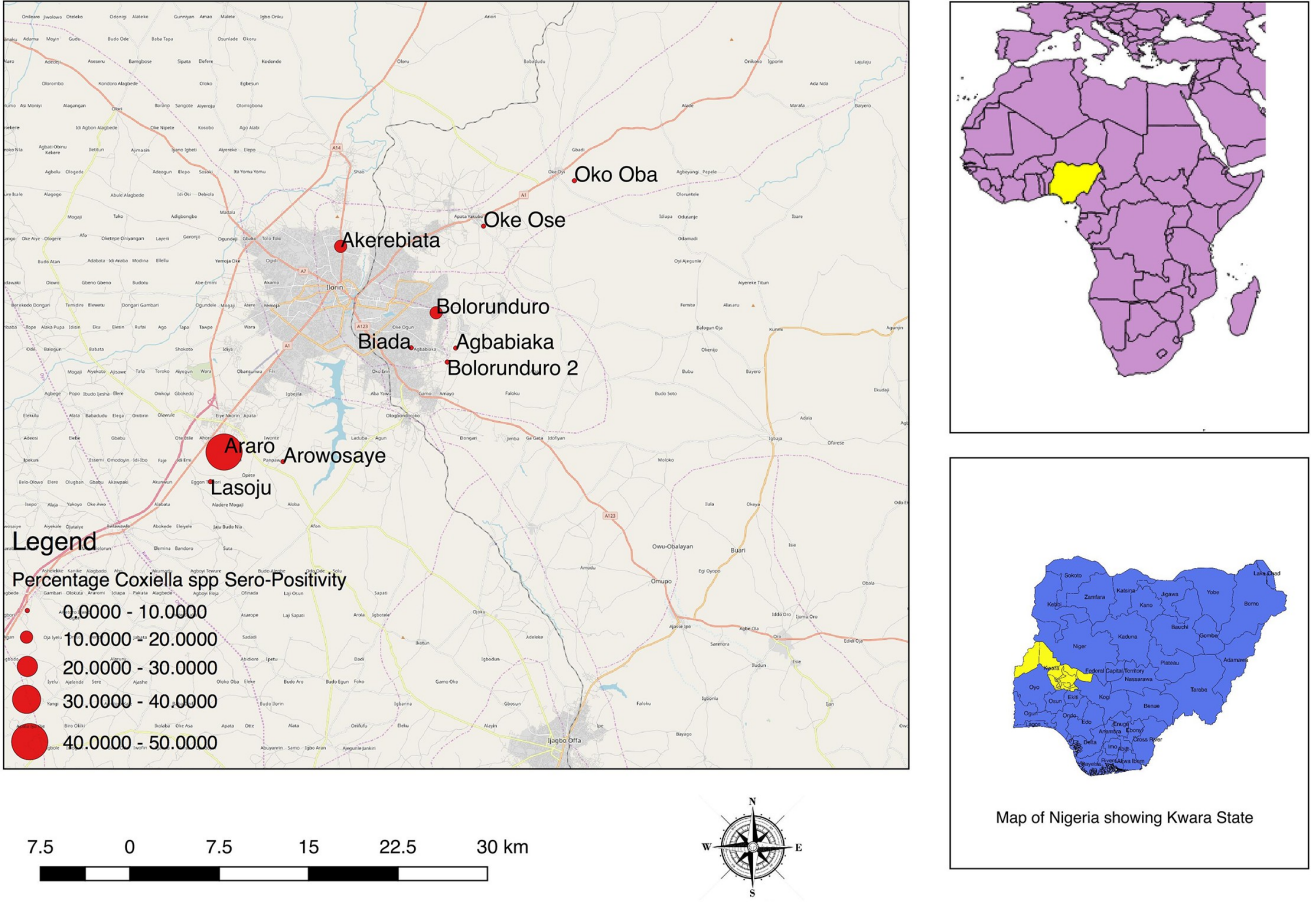
Map of villages sampled in Kwara State showing the proportion of cattle seropositive for *Coxiella burnetii*.

**Fig 3:**
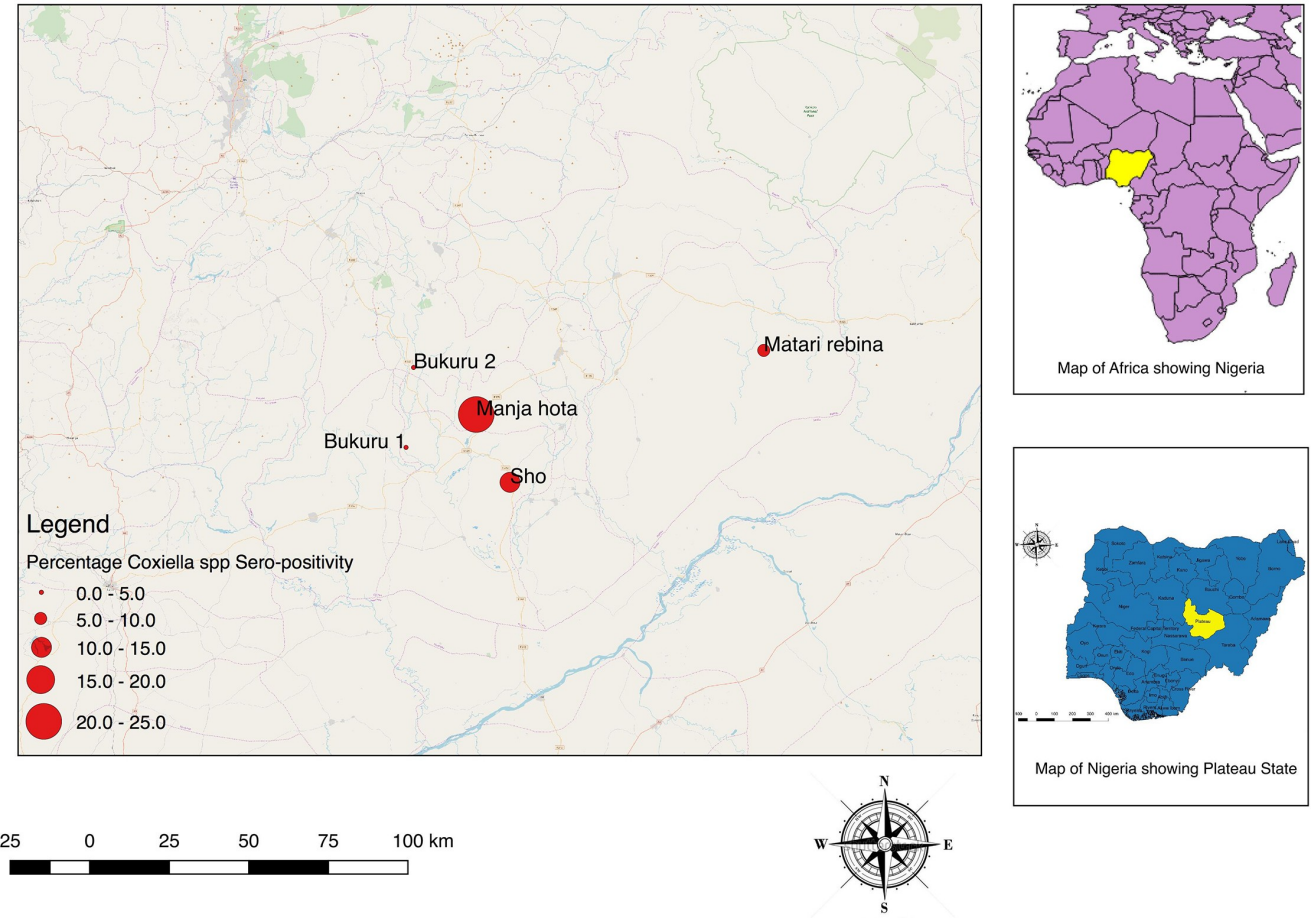
Map of villages sampled in Plateau State showing the proportion of cattle seropositive for *Coxiella burnetii*.

**Fig 4:**
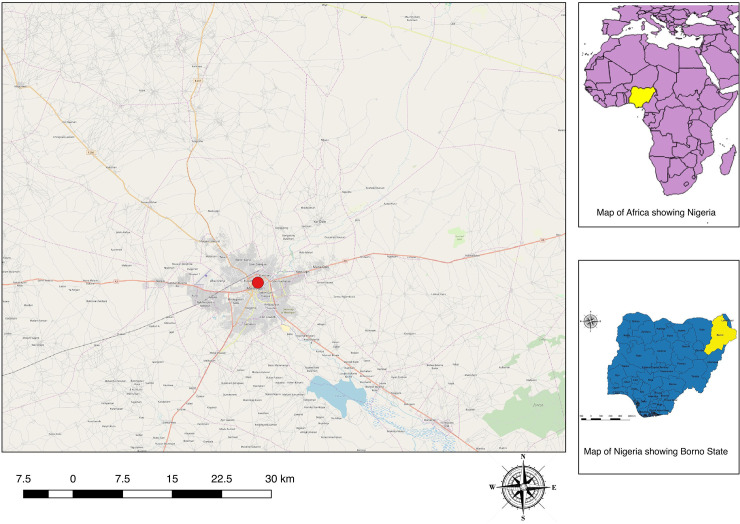
Map of Borno State showing the proportion of cattle seropositive for *Coxiella burnetii Coxiella burnetii* seropositivity.

The spatial distribution of sampled villages in Kwara, Plateau, and Borno states are shown in Figs 2–4 with the proportion of cattle seropositive for *Coxiella spp* is shown as proportional circles at each sampled village.

#### Cattle

The overall proportion of the sampled cattle that were positive for *C*. *burnetii* antibodies from Kwara State using ELISA was 14/90 (15.6%; 95% CI: 8.8–24.7). Seroprevalences at the village level ranged from 0.0% to 50.0% in cattle sampled from Kwara State (Table 2). The overall proportion of *C*. *burnetii* antibodies among cattle sampled from Plateau State using ELISA was 10/106 (9.43%; 95% CI: 4.6–16.7), while 4/72 (5.56%; 95% CI: 1.5–13.6) cattle serologically tested for *C*. *burnetii* from Borno State were positive.

**Table 2:** Bivariate analysis of possible risk factors with seroprevalence of *Coxiella burneti* in cattle sampled from households in Kwara State, northern Nigeria.

Variables	Number of cattle sampled	Number positive	Percentage positive %	P value
**Village**				
Agbabiaka	9	2	22.2	0.219
Akerebiata	20	3	15.0	
Araro	8	4	50.0	
Arowosaye	6	0	0.0	
Biada	6	0	0.0	
Bolonduro	19	5	26.3	
Lasoju	7	0	0	
Oke Ose	8	0	0	
Oko Oba	7	0	0	
**Age group**				
<1 year old	3	0	0	0.529
1–2 years old	16	1	6.3	
2–4 years old	40	8	20.0	
>4 years old	31	5	16.1	
**Sex**				
Male	16	2	12.5	0.710
Female	74	12	16.2	
**Breed of Cattle**				>0.999
White Fulani	80	12	15.0	
^λ^Other breeds	10	2	20.0	
*Presence of Ticks				0.378
Absent	13	1	17.5	
Present	57	10	7.7	
*Reproductive Status				
Lactating	34	6	17.6	0.813
Non-lactating	11	1	9.1	
Pregnant	3	1	33.3	
Active male	9	2	22.2	
Yearling	10	1	10.0	
Calves	3	0	0.0	
*Management System				
Extensive	57	11	19.3	0.226
Semi-intensive	7	0	0.0	
Intensive	6	0	0.0	

*20 cattle sampled from the abattoir were excluded.

λ –Other breeds include: Red Bororo, White Fulani Cross, Bokoloji, and Adamawa Cross.

Level of significance P < 0.05.

The results of the bivariate analysis (Table 2) calculated from cattle sampled from Kwara State, showed that the difference in *C*. *burnetii* seropositivity among the independent variables (village, age group, sex, breed of cattle, presence of ticks, reproductive status, and management system) was not statistically significant. Female (16.2%) cattle were slighter more exposed than male (12.5%). None of the cattle reared intensively and semi-intensive were seropositive, as all positive animals were from those reared extensively. However, this difference was not statistically significant. Seropositivity was found to be higher in older cattle aged between two and four years (8/40; 20%) and older than four years (5/31; 16.1%), though this difference was also not statistically significant (P = 0.529). Although more animals that were infested with ticks were seropositive, one of the cattle that was not infested with tick was also seropositive.

### Small ruminants

In Kwara State, a total of one hundred and eighty-four (184) small ruminants were sampled during the period of study. This was made up of 158 goats, and 26 sheep. The overall seroprevalence for *C*. *burnetii* using ELISA was 6/184 (3.3%; 95% CI: 1.2–7.0) among small ruminants sampled from Kwara State.

Out of 158 goats sampled, five (3.16%) were seropositive while only one out of 26 (3.85%) sheep was seropositive. More males (154) were slaughtered than females (30). However, a higher number of positives was recorded in female small ruminants slaughtered 2/30(6.67%) than males 4/154(2.59%).

The distribution of *C*. *burnetii* antibodies based on age group of small ruminants slaughtered showed that two out of the 80 young animals aged less than one year (2.5%) were seropositive; while four out of the 100 small ruminants aged between one and four years were seropositive (4.0%). However, none (0.0%) of the four animals aged four years or older were seropositive.

About 7.69% (13/184) of the small ruminants sampled at the abattoir were infested with ticks. However, only one of the 13 (7.69%) small ruminants that was infested with ticks tested positive for *C*. *burnetii* antibodies.

Furthermore, none of the possible predictors of infection among the small ruminants sampled showed statistically significant association with *Coxiella* seropositivity (Table 3). No significant difference (P < 0.05) was observed in the prevalence of *C*. *burnetii* antibodies in relation to species, sex, age group or presence of tick among the small ruminants sampled at the Ipata Municipal Abattoir, Ilorin.

**Table 3:** Bivariate analysis of possible risk factors with seroprevalence of *Coxiella burnetii* in small ruminants slaughtered at Ipata Municipal Abattoir, Kwara State, northern Nigeria.

Variables	Number of small ruminants sampled	Number positive	Percentage positive (%)	P value
**Age group**				
<1 year old	80	2	2.50	0.797
1–4 years old	100	4	4.00	
>4 years old	4	0	0.00	
**Sex**				
Male	154	4	2.59	0.251
Female	30	2	6.67	
**Small ruminant species**				
Caprine	158	5	3.2	0.856
Ovine	26	1	3.8	
**Breed of small ruminant**				
West African dwarf Goat	113	4	3.5	0.337
Sahel	38	0	0.0	
Sokoto Brown	7	1	14.3	
Yankassa	12	0	0.0	
Uda	3	0	0.0	
West African Dwarf Sheep	11	1	9.1	
**Presence of Ticks**				0.351
Absent	171	5	2.92	
Present	13	1	7.69	

Level of significance P < 0.05.

None of the 86 small ruminants sampled from Plateau State and assayed for *Coxiella* spp antibodies tested positive during the period of study.

## Discussion

### *Coxiella burnetii* seropositivity

To our knowledge, this is the first multi-state survey to determine the presence of antibodies to *C*. *burnetii* from both cattle and small ruminants in northern Nigeria. We found evidence of exposure to the pathogen in all the three states sampled. The percentage seropositivity to *Coxiella burnetti* in cattle (ranging from 5.56% - 15.6%) recorded from the present study is consistent with those reported from previous studies carried out among ruminants in other parts of Africa. In Nigeria, 14.5% of cattle were seropositive in one study [8], while another study reported 10%, 9% and 13% in goats, sheep, and cattle, respectively were seropositive [14]. Other studies in Egypt and Kenya reported 19.3% and 10.5% of cattle were seropositive [15, 16]. In Côte d’Ivoire, 13.9% of cattle, 9.4% of sheep, and 12.4% of goats were seropositive [27]. Testing for the presence of IgG antibody however indicates past exposure and not of current infection or shedding of *Coxiella* since animals may seroconvert without detectable shedding and can remain seropositive for years post-infection or sometimes may not seroconvert during active shedding of infection.

Findings from this study revealed a lower percentage seropositivity of 3.26% (6/184) for *C*. *burnetii* antibodies in small ruminants slaughtered from Kwara, while none of the small ruminants sampled from Plateau State tested positive. A recent study carried out to compare the presence of *C*. *burnetii* antibodies among animal species also reported lower percentages for sheep and goats [16]. It has been suggested that lower *Coxiella* seropositivity reported among goats especially meat goats compared to dairy goats might be due to the higher turnover of goats on meat farms, hence decreasing the time that goats are potentially exposed to the pathogen before the goats are slaughtered for meat [28]. This is certainly true for the goats sampled in this survey, as they were kept mainly for meat. On the other hand, similar studies carried out in The Gambia, a West African country, reported higher seroprevalence of *C*. *burnetii* antibodies of 18.5% in sheep and 24.2% in goats [29]. In another study carried out in Chad in Central Africa, seroprevalence rates of 11% and 13% were also reported in sheep and goats, respectively [29]. These values are slightly higher than those from the present study, but different literature show a wide variance in prevalence rates worldwide. For example, higher prevalence were reported in France (88.1%) and up to 40.0% in Bulgaria for goats. However, lower prevalence was reported from goats in Germany (2.5%) and Netherlands (7.8%). Similarly, variable prevalence has been recently reported for sheep: up to 56.9% in Bulgaria, 20.0% in France, 8.7% in Germany, 3.5% in the Netherlands [30].

The evidence of the exposure to *Coxiella* infection in both cattle and small ruminants is worrisome because, aside from its negative effect on reproductive performance such as abortion, stillbirth and reduced productivity of livestock, it is a persistent and highly transmissible pathogen to humans [5]. Moreover, pregnant, active male and lactating cows from the present study were more infected than cattle in other reproductive status from present study. *Coxiella burnetii* bacteria are excreted in bodily fluids including milk and milk products, which serves as the longest lasting source of human infection [7, 12, 31]. Infected cows are often asymptomatic but shed *C*. *burnetii* mainly in milk [5]. Cow milk is most commonly consumed by humans in the study area, either consumed whole or processed as “wara”, the local cheese, which maybe poorly pasteurised.

There was no statistically significant difference between the two species of small ruminant (P = 0.856) in the present study. A previous study, reported species of small ruminants to be a significant risk factor for *C*. *burnetii* infection with sheep appearing to have a significantly lower risk of being seropositive compared to goats [29]. Schelling et al. [32], also found prevalence to be higher in goats, while Ryan et al. [33] found prevalence to be higher in sheep. However, low numbers of sheep were available for sampling and this might affect the possibility of getting a greater seropositive animals. Sheep are kept for ceremonial occasions such as the Muslim Eid festivals and thus are rarely slaughtered any other period.

### Risk factors of exposure

None of the cattle aged less than one year from the present study was seropositive for *C*. *burnetii*. This differs from what we found in small ruminants, where both those less than 1 year and those aged between one and four years recorded seropositives. The differences in proportion of animals seropositive based on age group of both cattle and ruminants was however not significant. In contrast, other similar work Klaasen et al. [29], found that age of the animals appeared to be the most significant risk factor for seropositivity and they found that the older the animals were, the higher the risk of being seropositive. In their work, the difference was especially notable between animals younger than one year of age compared to older animals. Small ruminants between one to three years of age and animals four years or older were shown to be respectively 2.8 and 3.1 times more likely to be seropositive as compared to animals younger than one year of age [29]. The disparity between their findings and our findings is therefore most likely as a result of the fact that a large proportion of small ruminants sampled were below the age of one year (the most prevalent age for slaughter of small ruminants); and this might also be responsible for the low prevalence observed from our findings. Moreover only three cattle aged less than one year were sampled.

This study found that sex of animals was not a statistically significant risk factor of the seroprevalence of *C*. *burnetii* antibodies in both cattle and small ruminants sampled. The majority of the cattle kept in herds visited were female, with a few males kept mainly for servicing. On the other hand, the population of small ruminants sampled in the abattoir expectedly consisted of more males than females. This is because traditionally females are kept longer for breeding while males were slaughtered for meat. These two scenarios are likely to introduce gender bias in our statistical analysis and the result may not be a true reflection of lack of association. However, a past study from Nigeria found that the rate of infection was slightly higher among females than males and attributed this to the fact that the organism has a high affinity for the placenta, foetal membranes and mammary glands and is found in large numbers in these tissues [8].

Though the difference in seropositivity among breed of cattle sampled was found not to be statistically significant, a previous largescale study to determine the seroprevalence and risk factors of *Coxiella* infection reported that Friesian breed of cattle have increased odds of being infected than other breeds of cattle, although this is an Irish breed of cattle that is not common in Africa [34]. In the present study, the White Fulani were the predominant breed sampled and they were members of the same herd, thus may all have been served by same infected bull. Further investigation involving larger sampling of various animal breeds will provide conclusive information on breed association with *Coxiella* infection.

The absence of significant association between the presence of ticks on the body of cattle and small ruminants sampled and *C*. *burnetii* seropositivity is not unexpected. Studies have suggested that most tick species have low vector capacity to transmit *C*. *burnetii* and perhaps act only as secondary drivers of transmission compared to transmission via aerosols [35]. Although previous studies from Nigeria reported ticks to be infected with *Coxiella* spp. [9, 10], a recent study carried out in South Africa to detect tick-borne pathogens failed to detect *Coxiella* spp from a wide range of ticks tested by polymerase chain reaction (PCR) [36]. Even though several species of tick can be naturally infected with *C*. *burnetii*, they are however not important in the maintenance of infections in livestock or humans compared to wild vertebrates [37]. Furthermore, unlike other vector-borne pathogens, the presence of a vector such as ticks, bedbugs, flies and mites is not necessary for the transmission of the *C burnetii* from the reservoir to hosts [5]. Moreover, *C*. *burnetii* is highly infectious and is excreted in several bodily fluids including urine, milk, faeces, and birth products that contain large numbers of pathogens that become aerosolized and can be easily inhaled [38]. Aerosolized transport by wind of the spore-like pathogen has been reported to be the main route of introduction of *C*. *burnetii* on a farm [39]. In southeastern France, an area with high incidence *C*. *burnetii*, dissemination of spores by the local mistral wind is an important route of transmission between sheep herds breeding in the local plains. The author from that study speculated that the high endemicity may be related to aerosol contamination by the Mistral wind that blows through the local steppe [40]. Further evidence of spatial dispersal of *Coxiella* from infected livestock holding was also recently demonstrated [41].

The difference in seropositivity from different villages where cattle were sampled from Kwara State, was not statistically significant in the present study. However, a recent study carried out in Egypt, reported significant differences in animal seropositivity and domains [16]. It is suggested that further studies to determine the difference in specific geographical characteristics and climatic conditions on seroprevalence be carried out to establish the epidemiological reasons for these differences.

Results from this study showed that the types of management system did not seem to have a significant association between *Coxiella* seropositivity in cattle sampled. This finding was similarly reported in a previous study carried out in Zaria, Nigeria, that reported no difference in seropositivity for cattle reared semi-intensively versus those reared extensively [7]. Similar findings reported from Egypt also did not find any correlation between positive titres and husbandry practices [16].

## Conclusions

This study provides data on the current epidemiological situation of *C*. *burnetii* in cattle and small ruminants in the Northern Nigeria. Our findings further demonstrates the ubiquity of this pathogen as it is prevalent in all the areas studied.

Our ability to explore a range of predictors of seropositivity was limited by the incomplete data from seropositive animals in Plateau and Borno states. The fewer households covered may also introduce bias in the extensive study of risk factors of exposure to *Coxiella* spp. However, this does not negate the evidence of exposure from the study, since the disease is highly infectious, a single positive animal is a cause for concern.

Livestock keepers and stakeholders in animal health should be duly educated on proper disposal of birth products as well as bodily fluids in order to reduce environmental contamination and persistence.

One Health collaborative studies should be carried out to understand the epidemiological extent of human *Coxiella* infection in sub-Saharan Africa where the majority of residents depend on livestock for livelihood and where there are reports of fever of unknown origin.

## Supporting information

S1 Data (XLSX)
